# Artificial intelligence in healthcare faces regulatory challenges: balancing innovation with human oversight

**DOI:** 10.1016/j.ero.2026.02.010

**Published:** 2026-03-02

**Authors:** Herve Servy, Ben Braithwaite, Uta Kiltz, Astrid van Tubergen, Paul Studenic, Marieke Voshaar, Dieter Wiek, Sonia Tropé, Gerd Burmester, Vincenzo Venerito, Laure Gossec

**Affiliations:** 1 Sanoïa e-Health Services, Aubagne, France; 2 Rheumazentrum Ruhrgebiet, Herne, Ruhr-University Bochum, Bochum, Germany; 3 Department of Rheumatology, Maastricht University Medical Centre, Maastricht, The Netherlands; 4 Care and Public Health Research Institute, Maastricht University, Maastricht, The Netherlands; 5 Division of Rheumatology, Department of Internal Medicine 3, Medical University of Vienna, Vienna, Austria; 6 Department of Pharmacy and Department of Research & Innovation, Sint Maartenskliniek, The Netherlands; 7 Department of Pharmacy, Radboud University Medical Center, Nijmegen, The Netherlands; 8 EULAR PARE Patient Research Partner, Zurich, Switzerland; 9 Association Nationale de Défense contre la polyArthrite Rhumatoïde, Paris, France; 10 Department of Rheumatology and Clinical Immunology, Charité - University Medicine Berlin, Berlin, Germany; 11 Rheumatology Unit – Department of Precision and Regenerative Medicine and Ionian Area, University of Bari “Aldo Moro”, Bari, Italy; 12 Sorbonne Université, INSERM, Institut Pierre Louis d’Epidémiologie et de Santé Publique, Team Pepites, Paris, France; 13 AP-HP, Pitié-Salpêtrière Hospital, Rheumatology Department, Paris, France

## Abstract

Artificial intelligence (AI) has experienced unprecedented growth in the healthcare sector, promising to revolutionise diagnosis, care, treatment, and biomedical research. This viewpoint article discusses the challenges in this sector and highlights the evolution of the regulatory framework surrounding AI in healthcare and its concrete consequences on innovation. Layered regulatory developments, notably the Medical Device Regulation of 2021 and the AI Act of 2024, have introduced stricter requirements for traceability, clinical performance, risk management, transparency, and human oversight. Although these changes are expected to foster greater trust and accountability among healthcare stakeholders, they also increase the complexity and cost of compliance. These regulatory and organisational challenges represent a turning point for innovative projects in healthcare. Many projects are moving from complex systems toward simpler human-centred tools, whereas others are pushing for collaboration between stakeholders to harmonise existing regulatory frameworks. These combined efforts could lead to more compliant, understandable, and user-friendly solutions for routine care.

## INTRODUCTION

In the 2010s, the healthcare sector experienced an unprecedented boom in research on the development and clinical applications of systems powered by artificial intelligence (AI). Driven by advances in machine learning, the growing diversity and availability of medical data, and support from public and private investment, a wave of innovations aimed to revolutionise diagnosis, care, treatment, and biomedical research. This enthusiasm led to a proliferation of pilot projects, specialised start-ups, and partnerships between medical institutions and technology companies [1,2].

It was against this backdrop of excitement that we launched an innovative study named ActConnect in 2016 [3]. In this pilot study, we applied machine learning techniques to passive physical activity data from wearable technology (an activity tracker watch), aiming to detect disease flares in inflammatory arthritis [3,4]. The AI model, developed on minute-level step count data from 174 patients with rheumatoid arthritis or axial spondyloarthritis, was able to differentiate self-reported flares from nonflare weeks extremely well (sensitivity and specificity >94%) [4,5]. Based on these findings, we planned a much wider-scale, international project (called a personalised, dynamic APpointment PRIoritisation SystEm using data from wearables processed by AI for patients with inflammatory arthritis (APPRISE)) to demonstrate the feasibility of a just-in-time intervention based on AI-detected disease flares, as determined using activity data (Box) [3,4,6].

Box **The APPRISE project: an example of a cancelled project**

**Table:** 

The APPRISE study was designed as an ambitious international randomised controlled unblinded pragmatic trial aiming to follow 800 patients with rheumatoid arthritis, axial spondyloarthritis or psoriatic arthritis over 1 year. It was awarded a grant from FOREUM (Foundation for Research in Rheumatology) [3,6]. All patients would wear an activity tracker. Flares were to be detected using an artificial intelligence (AI) algorithm measuring changes in walking patterns. The intervention group would have a run-in period where they would self-report flares weekly to calibrate the AI algorithm. The initial algorithm was planned to be redeveloped using the anonymised data from the previously published ActConnect study (original algorithm not publicly available) [3]. In the next step, follow-up visits would be scheduled according to the AI-detected flares. The control group would receive usual care. This ambitious study was developed based on the findings of a pilot study performed a few years ago, exploring the link between steps and flares, the ActConnect study [4]. However, the APPRISE project had to be discontinued, following changes in the regulatory landscape, as well as technical difficulties.

Alt-text: Unlabelled box dummy alt text

However, the landscape of AI-powered research is changing quickly, and this ambitious project has been cancelled, faced with new technical and regulatory hurdles.

Drawing from this experience, we analyse the main regulatory changes of recent years regarding AI, their impact on AI projects in healthcare [7], and call for further collective reflection on how highly innovative technological advances, including AI tools, can be researched, developed, and validated for routine clinical use in an evolving regulatory landscape.

## REGULATORY DEVELOPMENTS: FROM VAGUENESS TO COMPLEXITY

In 2016 and 2017, at the time of our original ActConnect study (Box), digital health tools, including those incorporating AI, were operating in a regulatory grey area due to the degree of novelty in their approach. CE (Conformité Européenne [European Conformity]) marking for software medical devices (SMDs) remained relatively unrestrictive, and there were few requirements relating specifically to AI.

The major turning point came in 2021, with the enforcement of Regulation (European Union [EU]) 2017/745 on medical devices (known as the Medical Device Regulation [MDR]) [8], which overhauled the obligations for medical device manufacturers, including medical software developers. The new requirements outlined the need for increased traceability, demonstration of clinical performance, continuous postmarket evaluation, rigorous risk management, and enhanced technical documentation [9]. In most cases, AI tools require high resources and a rigorous and complex assessment process, on top of an evaluation procedure by a notified body [10,11].

Finally, between 2022 and 2025, the EU embarked on further regulatory restructuring through the development of the AI Act (AI Act) [12]. The AI Act applies to systems placed on the market, put into service, or used in the EU, and introduces a risk-based classification of AI systems. Medical AI systems destined for widespread real-world use are considered ‘high-risk’, meaning developers of such systems must request third-party conformity assessment under the MDR, and imposing additional requirements for transparency, explainability, and human oversight. However, ‘in-house’ devices developed and used within healthcare institutions, and thus not meant to be distributed (MDR Art. 5(5)), are excluded from this high-risk category [8]. Notably, these include large language models (LLMs) run locally within a hospital to support note drafting or diagnostic procedures.

Generally, the AI Act does not regulate research or testing activities in themselves and excludes research and testing before placing on the market. However, real‑world testing is allowed only after demonstrating that the device meets the Act’s approval/registration and safeguard requirements (Art. 60), or by operating inside a regulatory sandbox (ie, a strictly supervised experimentation environment as defined in Art. 57) [12].

Thus, the MDR and AI Act layer into each other and present complex challenges for AI as part of a medical device. The Medical Device Coordination Group (MDCG) provides guidance for how to apply these regulations [10]. In particular, the 2025 MDCG/Artificial Intelligence Board joint guidance (MDCG 2025‑6) explains how high‑risk AI must generate clinical evidence under MDR/in vitro diagnostic regulation and how AI‑Act obligations layer in [13].

Overall, these developments, while necessary to regulate a technology with significant potential impact, have produced a highly complex regulatory environment and profoundly changed the landscape of healthcare innovation, which sponsors must learn to navigate (Table).

**Table:** Summary of the regulatory landscape for AI including the MDR, MDCG Guidelines [8], and the AI Act [10]

Regulatory aspect	Medical Device Regulation (MDR - EU 2017/745)	MDCG guidelines	AI Act
Main objective	To strengthen the safety and performance of MDs, harmonise rules within the EU, and increase transparency.	To provide practical and harmonised guidance for the application of the MDR. These guides are not legally binding but represent the common position of competent authorities.	To establish a harmonised legal framework for the development, market placement, and use of AI systems, based on a risk-based approach.
Scope	Applies to all medical devices placed on the European market, including software and products without an explicit medical purpose but with similar characteristics (Annex XVI).	Covers a wide range of topics related to the MDR, such as MD classification, clinical evaluation, postmarket surveillance, cybersecurity, etc.	Applies to all AI systems placed on the market or put into service in the EU. MDs incorporating AI are directly concerned.
Classification	Maintains a risk-based classification system (Class I, IIa, IIb, III), but with stricter rules, particularly for software (Rule 11), which is often reclassified into a higher risk category.	Offers clarifications and examples to help manufacturers correctly apply the MDR's classification rules, especially for borderline products and software (eg, MDCG 2021-2024).	Classifies AI systems based on their risk level (unacceptable, high, limited, minimal). Most MDs integrating AI are classified as ‘high-risk’, which triggers additional requirements.
Key contents/requirements	- Strengthened clinical evaluation: More robust clinical evidence is required. - Postmarket Surveillance (PMS): Implementation of a proactive system for data collection and analysis. - Unique Device Identification (UDI): Mandatory traceability system. - Increased transparency: EUDAMED database to centralise information (by 2027). - Economic operators: Clarified obligations for manufacturers, authorised representatives, importers, and distributors.	- Guidance on clinical evaluation: Details on required evidence and equivalence. - Guidance on classification: Help in interpreting classification rules. - Guidance on cybersecurity: Recommendations for protection against cyber threats (eg, MDCG 2019-2016). - Guidance on "significant changes": Criteria to determine if a modification to a device under the old directive requires a new MDR certification.	- Quality and risk management system: Specific to AI-related risks. - Data quality and governance: Requirements for training, validation, and testing datasets. - Technical documentation: Must demonstrate compliance with AI Act requirements. - Transparency and user information. - Human oversight: Measures to allow for adequate human supervision.
Complementarity	The MDR constitutes the regulatory foundation for placing MDs on the market. It acknowledges the importance of software and programmable electronic system safety.	MDCG guidelines are essential for interpreting and implementing MDR requirements in a harmonised way. They provide concrete examples and clarifications expected by the industry.	The AI Act adds an additional regulatory layer for MDs that are or incorporate AI systems. A "high-risk" MD under the AI Act will need to comply with both the MDR and the AI Act. Conformity assessment procedures will be required.
Enforcement timeline	Progressive enforcement since May 26, 2021, with extended transition periods until 2027 or 2028 depending on the device's risk class.	Published continuously to support the implementation of the MDR.	Adopted in 2024, to be progressively applied over the following 2 years.

AI, artificial intelligence; EU, European Union; EUDAMED, European Database on Medical Devices; MDCG, Medical Device Coordination Group; MD, medical device; MDR, Medical Device Regulation; PMS, Postmarket Surveillance; UDI, unique device identification.

## THE IMPACT OF REGULATORY CHANGES

Tightening the regulatory framework is having direct and sometimes decisive consequences for AI projects in healthcare.

### Noncompliant or insufficient data

The MDR outlines rigorous requirements for anonymisation, traceability, quality, and labelling of data, which are closely aligned with patients’ and citizens’ expectations in terms of privacy, control over secondary use of their data, and protection against reidentification. However, building a substantial and fully compliant dataset can be complex, especially for multicentre trials, requiring a harmonised workflow across all participating centres [14,15]. For instance, the *AI4HI* projects (AI for Health Imaging) faced significant challenges due to centre-dependent variability in anonymisation and data standardisation practices [16]. These required a complete redefinition and harmonisation of data deidentification workflows, leading in some cases to the redesign of data collection pipelines to ensure compliance with ethical and legal standards [16,17].

### Clinical demonstration hindered by regulatory and operational constraints

Demonstrating the clinical performance of an AI algorithm across diverse healthcare settings and over time is slowed by the complexity of evaluation processes. Conducting prospective studies that meet the requirements of EU regulations demands not only a rigorous methodology but also significant, yet frequently limited, financial and human resources [18]. For instance, the *EuCanImage* project, which aims to build a large-scale European cancer imaging platform, encountered delays due to data-sharing procedures and cross-border ethical approvals [19]. Though these are not strictly speaking new hurdles, the newly reinforced review processes tend to accentuate them.

### Compliance costs

Achieving full regulatory compliance, including certification fees and technical file preparation, can be expensive, and may exceed initial estimates of projects developed under the previous framework. Therefore, projects transitioning from 1 framework to another can face extra costs, which may prove prohibitive [14,20].

### Mistrust from care structures, healthcare professionals, and patients

Despite encouraging retrospective performance, hospital authorities, healthcare professionals, and patients have expressed reservations—driven in part by limited familiarity with AI and concerns about transparency, interpretability, and risk of excessive automation [18,21]. A 2025 survey of 778 rheumatology patients in Germany highlights this duality of perceptions surrounding AI use. Authors found that, while 57.6% of patients welcomed the use of AI for health-related purposes, a significant proportion remained sceptical, with 37.9% reporting not knowing about AI at all [22].

Recent regulations partly address these concerns by strengthening transparency, traceability, data quality, and anonymisation requirements, and by introducing an explicit requirement for AI literacy for providers and deployers (AI Act, Article 4 [12]), intended to support responsible use and user confidence. Importantly, many patient organisations emphasise that trust also depends on early and continuous patient involvement in the design, governance, and communication surrounding AI tools—an essential complement to regulatory safeguards.

### How did these regulations affect the APPRISE study?

The APPRISE project (Box) illustrates the impact of the evolving regulatory landscape on high-risk medical AI. Although the MDR technically allows clinical investigations of SMDs without a CE mark to generate evidence, the reclassification of software as higher risk under the MDR, combined with the AI Act’s designation of medical AI as high-risk, has created a substantial barrier. For APPRISE, the volume of data, traceability, and risk-management documentation required to obtain authorisation for a large-scale trial was close to what was needed for the final CE mark itself. As a result, what had been feasible under the previous regulatory “grey area” became unworkable, leading to the project’s discontinuation. This example highlighted how stringent new requirements for high-risk AI could hinder real-world clinical testing despite promising technology.

Faced with these challenges, many other projects have revised their objectives. Some have updated their methodology at the cost of delays [19], while others have preferred simpler decision-making rules, with projects being reoriented towards more understandable tools for caregivers or patients [23].

The limitations placed on innovation in Europe should also be seen in the light of progress being made in other parts of the globe. Specifically, in rheumatology and in healthcare more generally, it might be useful for EULAR and the Biomedical Alliance to develop a ‘call for action’.

## MOVING (BACK) TO A MORE ‘HUMAN-CENTRIC’ APPROACH

In recent years, regulatory uncertainty and complexity have led several ambitious AI-driven projects to be suspended or redirected toward simpler, more readable, more user-centred tools [24,25]. These alternatives prioritise data management, visualisation, user experience, and timely data transmission, rather than the demanding technical requirements of high-risk AI systems. As a result, we are witnessing a renewed interest in established clinical tools—validated scores, checklists, transparent decision trees, and personalised yet nonautomated recommendations—which often align more closely with current expectations around explainability, ethics, and accountability. In parallel, some teams are developing “low-tech” or narrowly focused AI models co-designed with clinicians to ensure usability, appropriate oversight, and integration into clinical workflows [23].

Another approach to human-centricity in clinical AI is evidenced by the choice of implementation strategies, avoiding the “high-risk” classification. For instance, under MDR Article 5(5) [8], the use of locally hosted LLMs is permitted within hospital systems. A purely local implementation enables institutions to process sensitive data within secure health data systems while ensuring clinician oversight, thus ensuring a human-centric decision-making process while achieving favourable performance. Indeed, a recent benchmarking study found that locally deployed LLMs had the potential to outperform traditional diagnostic support systems for identifying rare rheumatic diseases [26].

A complementary approach involves integrating electronic patient-reported outcomes and digital biomarkers into clinical workflows. This could enhance clinician-patient communication and foster patient participation, in accordance with the AI Act’s focus on AI literacy (Article 4) [12].

Collectively, these components demonstrate that a human-centric approach to the development and use of AI is evolving into a regulatory and structural necessity. However, this comprehensive approach mandates a solid budget, a multidisciplinary team and a robust infrastructure for successful testing. Ultimately, beyond these technical and regulatory considerations, whether advanced or simple, AI should remain a support for clinicians’ judgement—not a substitute for it.

## CONCLUSION

The initial euphoria surrounding AI in healthcare has collided, sometimes brutally, with regulatory and ethical realities. Although stricter regulatory requirements are essential to ensure safety, transparency, and quality of care, they have also exposed a gap between the potential of AI systems and the practical conditions for implementing them in real-world clinical settings. However, rather than signalling a setback, this tension highlights a critical phase of maturation for AI in healthcare which concerns all stakeholders.

Recent studies clearly identify collaboration between researchers, funders, patients, and professional societies as key to overcoming the hurdles and complexity we outlined in this viewpoint. Indeed, a position paper in gastro-enterology presents practical recommendations for AI use in hepatology—many of which could be adapted for rheumatology—highlighting the need for harmonised regulatory requirements, standardised data systems, validated evaluation methods, and updates to clinical practice guidelines [27]. These conclusions are further supported by a recent qualitative survey, wherein stakeholders in digital health technologies identified regulatory complexity as a key barrier for the implementation of early feasibility studies [28]. This problem disproportionately affects smaller teams, underscoring the value of collaborative initiatives such as the Harmonized Approach for Early Feasibility Studies for Medical Devices in the EU, which aim to draw from existing programmes to simplify European frameworks and facilitate innovation.

Many hurdles still remain, but, by drawing on experience from past projects and other, simpler frameworks, the scientific community is collaborating to move towards a more harmonious regulatory landscape, paving the way for even more innovative projects and helping them avoid pitfalls. We expect these efforts will help facilitate the development of human-centred AI tools and provide opportunities to deliver systems that are more grounded in clinical realities, accountable, implementable, and ultimately more beneficial for patients and healthcare professionals alike.

## CRediT authorship contribution statement

**Herve Servy:** Supervision, Methodology. **Ben Braithwaite:** Writing – review & editing, Writing – original draft, Methodology, Conceptualization. **Uta Kiltz:** Writing – review & editing, Methodology, Conceptualization. **Astrid van Tubergen:** Writing – review & editing, Methodology, Conceptualization. **Paul Studenic:** Writing – review & editing, Methodology, Conceptualization. **Marieke Voshaar:** Writing – review & editing, Methodology, Conceptualization. **Dieter Wiek:** Writing – review & editing, Methodology, Conceptualization. **Sonia Tropé:** Writing – review & editing, Methodology, Conceptualization. **Gerd Burmester:** Writing – review & editing, Methodology, Conceptualization. **Vincenzo Venerito:** Writing – review & editing, Methodology, Conceptualization. **Laure Gossec:** Writing – review & editing, Writing – original draft, Validation, Supervision, Project administration, Methodology, Funding acquisition.

## Competing interests

LG reports financial support from FOREUM. LG has received consulting or advisory fees, funding grants, non-financial support, speaking and lecture fees, and/or travel reimbursement from AbbVie Inc, Eli Lilly and Company, Novartis, UCB, Alfasigma, Bristol-Myers Squibb Company, Celltrion, Inc., Johnson and Johnson, MoonLake Immunotherapeutics Ltd, Oruka Therapeutics Inc, Pfizer, Takeda, Amgen Inc, Stada, and Biogen. LG also reports board membership with the European Alliance of Associations for Rheumatology. UK reports consulting or advisory fees and funding grants from AbbVie Inc, Alfasigma USA, Inc., Amgen Inc, Biogen Inc, Bristol-Myers Squibb Company, Celltrion, Inc., Chugai Pharmaceutical Co Ltd, Eli Lilly and Company, Fresenius Medical Care Holdings Inc, GSK, Johnson and Johnson, MSD, Novartis, Pfizer, Roche, and UCB. UK also reports board membership with the European Alliance of Associations for Rheumatology and the National Rheumatology Board. AvT reports consulting or advisory fees, funding grants, and speaking and lecture fees from Novartis; consulting or advisory fees and funding grants from UCB; consulting or advisory fees from Johnson and Johnson; and speaking and lecture fees from Pfizer. PS reports speaking and lecture fees from Bristol-Myers Squibb Company, CSL Vifor, and AbbVie Inc; travel reimbursement from Johnson and Johnson; and a relationship with AstraZeneca. GB reports consulting or advisory fees and speaking and lecture fees from AbbVie Inc, ADVANZ PHARMA Corp., Amgen Inc, Biogen, Gilead Sciences Inc, Eli Lilly and Company, MSN Group, Novartis, Pfizer, Sandoz Inc, Sanofi, and UCB. VV reports consulting or advisory fees, funding grants, and speaking and lecture fees from Eli Lilly and Company, Novartis, UCB, GSK, and Pfizer; speaking and lecture fees from AbbVie Inc, Janssen, and Boehringer; funding grants and speaking and lecture fees from Galapagos and Alfasigma; and board membership with the European Alliance of Associations for Rheumatology and the Italian National Rheumatology Board. All other authors declare no known competing financial interests or personal relationships that could have appeared to influence the work reported in this paper.

## References

[1] Powles J., Hodson H. (2017). Google DeepMind and healthcare in an age of algorithms. Health Technol (Berl).

[2] Jimma BL. (2023 Mar 1). Artificial intelligence in healthcare: a bibliometric analysis. Telemat Inform Rep.

[3] Gossec L., Guyard F., Leroy D., Lafargue T., Seiler M., Jacquemin C. (2019 Oct). Detection of flares by decrease in physical activity, collected using wearable activity trackers in rheumatoid arthritis or axial spondyloarthritis: an application of machine learning analyses in rheumatology. Arthritis Care Res (Hoboken).

[4] Jacquemin C., Molto A., Servy H., Sellam J., Foltz V., Gandjbakhch F. (2017 June 29). Flares assessed weekly in patients with rheumatoid arthritis or axial spondyloarthritis and relationship with physical activity measured using a connected activity tracker: a 3-month study. RMD Open.

[5] Jacquemin C., Servy H., Molto A., Sellam J., Foltz V., Gandjbakhch F. (2018 Jan 2). Physical activity assessment using an activity tracker in patients with rheumatoid arthritis and axial spondyloarthritis: prospective observational study. JMIR MHealth UHealth.

[6] FOREUM grants [Internet]. Foreum. [cited 2025 Oct 1]. Available from: https://foreum.org/foreum-grants/

[7] Aboy M., Minssen T., Vayena E. (2024 Sept 6). Navigating the EU AI Act: implications for regulated digital medical products. NPJ Digit Med.

[8] European Union. Regulation (EU) 2017/745 of the European Parliament and of the Council of 5 April 2017 on medical devices. Luxembourg: Publications Office of the European Union; 2017 [cited 2025 Oct 1]. Available from:https://eur-lex.europa.eu/legal-content/EN/TXT/?uri=CELEX%3A02017R0745-20260101EUR-Lex-02017R0745-20250110

[9] VDE. The EU Medical Device Regulation (MDR): What changes? [Internet]. Offenbach am Main: VDE; 2024. [cited 2025 Oct 1]. Available from:https://www.vde.com/topics-en/health/consulting/the-eu-medical-device-regulation-mdr-what-changes?

[10] European Commission. MDCG endorsed documents and other guidance.Brussels: European Commission; 2025. [cited 2025 July 29]. Available from:https://health.ec.europa.eu/medical-devices-sector/new-regulations/guidance-mdcg-endorsed-documents-and-other-guidance_en

[11] Gerhart M. (2025). MDR Classification Rule 11: The classification nightmare? [Internet]. Regulatory knowledge for medical devices.

[12] European Union. Regulation (EU) 2024/1689 of the European Parliament and of the Council of 13 June 2024 laying down harmonised rules on artificial intelligence and amending Regulations (EC) No 300/2008, (EU) No 167/2013, (EU) No 168/2013, (EU) 2018/858, (EU) 2018/1139 and (EU) 2019/2144 and Directives 2014/90/EU, (EU) 2016/797 and (EU) 2020/1828 (Artificial Intelligence Act). Luxembourg: Publications Office of the European Union; 2024 [cited 2025 Oct 1]. Available from: http://data.europa.eu/eli/reg/2024/1689/oj/eng

[13] Medical Device Coordination Group (MDCG). MDCG 2025-6: FAQ on Interplay between the Medical Devices Regulation & In vitro Diagnostic Medical Devices Regulation and the Artificial Intelligence Act [Internet]. Brussels: European Commission; 2025. [cited 2025 Nov 21]. Available from:https://health.ec.europa.eu/latest-updates/mdcg-2025-6-faq-interplay-between-medical-devices-regulation-vitro-diagnostic-medical-devices-2025-06-19_en

[14] Joswig T, Kurz W. Regulatory and Compliance Requirements for SMEs Operating AI Systems through Data Centers in the EU, with a Focus on Data Protection Challenges in Germany. *Journal of Next-Generation Research 5.0*, 2025 [cited 2025 Aug 5]; Available from: https://jngr5.com/jngr/article/view/89

[15] Biasin E., Kamenjašević E., Ludvigsen KR., Solaiman B., Cohen I.G. (2024). Research Handbook on Health, AI and the Law [Internet].

[16] Pesapane F., Hauglid M.K., Fumagalli M., Petersson L., Parkar A.P., Cassano E. (2025). The translation of in-house imaging AI research into a medical device ensuring ethical and regulatory integrity. Eur J Radiol.

[17] Kondylakis H., Catalan R., Alabart S.M., Barelle C., Bizopoulos P., Bobowicz M. (2024 May 31). Documenting the de-identification process of clinical and imaging data for AI for health imaging projects. Insights Imaging.

[18] Behzad S., Tabatabaei S.M.H., Lu M.Y., Eibschutz L.S., Gholamrezanezhad A. (2024 Oct). Pitfalls in interpretive applications of artificial intelligence in radiology. AJR Am J Roentgenol.

[19] EuCanImage successfully closes first reporting period [Internet]. EuCanImage. [cited 2025 Aug 6]. Available from: https://eucanimage.eu/eucanimage-successfully-closes-first-reporting-period/.

[20] Becker K., Lipprandt M., Röhrig R., Neumuth T. (2019 Oct 1). Digital health – Software as a medical device in focus of the medical device regulation (MDR). It - Inf Technol.

[21] Pesapane F., Rotili A., Valconi E., Agazzi G.M., Montesano M., Penco S. (2023 Jan 1). Women’s perceptions and attitudes to the use of AI in breast cancer screening: a survey in a cancer referral centre. Br J Radiol.

[22] Labinsky H., Klemm P., Graalmann L., Thiele T., Hornig J., Fink D. (2025 Nov 10). Patient experiences, attitudes, and profiles regarding artificial intelligence in rheumatology: a German national cross-sectional survey study. Rheumatol Int.

[23] Manju, V.N., and N. Aparna. “Decision tree-based explainable AI for diagnosis of chronic kidney disease.” 2023 5th International Conference on Inventive Research in Computing Applications (ICIRCA). IEEE, 2023. [cited 2026 Feb 26]. Available from: https://ieeexplore.ieee.org/document/10220774.

[24] Olesch A. Does Forward Health's failure mark the winter of telehealth? In : *ICT&health* [online article], 2024. [cited 2025 Dec 10]. Available from:https://www.icthealth.org/news/does-forward-healths-failure-mark-the-winter-of-telehealth

[25] Babylon Health: the failed AI wonder app that “dazzled” politicians [Internet]. The Week; 2023 [cited 2025 July 29]. Available from: https://theweek.com/health/babylon-health-the-failed-ai-wonder-app-that-dazzled-politicians

[26] Kremer P., Schiebisch H., Lechner F., Haase I., Cirkel L., Kötter I. (2025 June 1). Comparative analysis of large language models and traditional diagnostic decision support systems for rare rheumatic disease identification. EULAR Rheumatol Open.

[27] Clusmann J., Balaguer-Montero M., Bassegoda O., Schneider C.V., Seraphin T., Paintsil E. (2025 Dec). The barriers to uptake of artificial intelligence in hepatology and how to overcome them. J Hepatol.

[28] Peseke M., Michaelis I., Kayembe O.T., Geraghty M., McDonnell A., Zurlo F.L. (2025 Oct 1). Stakeholder perspectives on early feasibility studies for digital health technologies in the European Union: qualitative interview study. J Med Internet Res.

